# Metabolic, cognitive and neuromuscular responses to different multidirectional agility-like sprint protocols in elite female soccer players – a randomised crossover study

**DOI:** 10.1186/s13102-024-00856-y

**Published:** 2024-03-06

**Authors:** Christian Raeder, Meike Kämper, Arthur Praetorius, Janina-Sophie Tennler, Christian Schoepp

**Affiliations:** 1 Department of Arthroscopy Surgery, Sports Traumatology and Sports Medicine, BG Klinikum Duisburg, Duisburg, Germany; 2 Women´s Soccer Department, MSV Duisburg, Duisburg, Germany; 3 Department of Trauma and Reconstructive Surgery, University Hospital Essen, Essen, Germany

**Keywords:** Agility, Sport-specific fatigue, Repeated-sprint exercise, Soccer, Female athletes, Return to sports, Performance testing, Sports injury, Sports medicine, Sports rehabilitation

## Abstract

**Purpose:**

Resistance to fatigue is a key factor in injury prevention that needs to be considered in return-to-sport (RTS) scenarios, especially after severe knee ligament injuries. Fatigue should be induced under game-like conditions. The SpeedCourt (SC) is a movement platform for assessing multidirectional sprint performance, typical of game-sports, due to change-of-direction movements in response to a visual stimulus. Designing adequate fatigue protocols requires the suitable arrangement of several loading variables such as number of intervals, sprint distance or work/relief ratio (W:R). Therefore, this study analysed the acute fatigue effects of different SC protocols on metabolic load, cognitive function and neuromuscular performance.

**Methods:**

Eighteen female soccer players (mean ± SD; age: 23.1 ± 4.6 years) of the 1st German Division participated in this randomised, crossover study. Using a random allocation sequence, players completed four volume-equated protocols differing in W:R and sprint distance per interval (P1:12 × 30 m, W:R = 1:2 s; P2:12 × 30 m, W:R = 1:3 s; P3:18 × 20 m, W:R = 1:2 s; P4:18 × 20 m, W:R = 1:3 s). Pre- and post-exercise, metabolic load was measured per blood lactate concentration (BLaC), cognitive function per reaction time (RT), and neuromuscular performance including multiple rebound jumps (MRJ height, primary outcome variable; Reactive Strength Index, RSI) and 5 m sprint times (SP5).

**Results:**

Repeated-measures ANOVA revealed significant main time effects (*p* < .05) with improved performance post-exercise in RT (504 vs. 482 ms, d = 1.95), MRJ height (24.0 vs. 24.8 cm, d = 0.77), RSI (1.39 vs. 1.43, d = 0.52), and SP5 (1.19 vs. 1.17 s, d = 0.56). There was significant main time (*p* < .001) and time x protocol interaction effects in BLaC (*p* < .001). P1 induced higher BLaC values (4.52 ± 1.83 mmol/L) compared to P2 (3.79 ± 1.83 mmol/L; d = 0.74) and P4 (3.12 ± 1.83 mmol/L; d = 1.06), whereas P3 (4.23 ± 1.69 mmol/L) elicited higher BLaC values compared to P4 (d = 0.74).

**Conclusion:**

All protocols caused an improved cognitive function and neuromuscular performance. The former may be related to enhanced noradrenergic activation or exercise specificity which induced an improved stimulus processing. The latter may be explained by a possible post-activation performance enhancement effect on jump and sprint performance. A shorter relief duration in W:R as opposed to sprint distance per interval produced higher BLaC values. The protocols may serve as reference data for improved RTS decision-making in elite female soccer players.

**Trial registration:**

Deutsches Register Klinischer Studien (DRKS), No.: DRKS00033496, Registered 19. Februar 2024, Retrospectively Registered.

## Introduction

Field-based game sports such as soccer or handball are characterized by an intermittent activity profile with a common stop-and-go pattern of play [1, 2]. Game sport athletes frequently engage in short high-intensity or maximal running efforts (e.g., sprints, accelerations), interspersed with brief recovery periods of rest or submaximal intensity [3, 4]. A high level of aerobic fitness combined with an optimal fatigue resistance is required in order to maintain power output and to recover quickly between repeated high-intensity efforts [1, 5]. During intermittent efforts, players execute a wide range of changes of direction (CODs) at various angles that include twisting, turning or cutting manoeuvres, [6, 7]. These explosive COD actions are rarely pre-planned or specifically rehearsed movement patterns, but are performed contextually in response to an external stimulus for a game-related purpose, such as quickly evading an opponent or reacting to a moving ball [1, 8]. This is referred to as agility, which encompasses unplanned open-skill tasks during match play, taking into account the perceptual-decision making process at the cognitive level (e.g., visual scanning, attentional focus, pattern recognition) and its physical outcome, a reactive COD or change in speed [9, 10].

In addition to being a key ability associated with success in multidirectional sports, CODs are also key actions linked to lower limb injuries such as an anterior cruciate ligament (ACL) tear [6]. In women´s soccer, ACL injuries account up to one third of all injuries per season [11] with 2.2 ACL injuries per 1000 h of match play [12]. Furthermore, female soccer players have a 2–3 times higher ACL injury risk compared to their male counterparts [13]. Typical test batteries for return-to-sport (RTS) after ACL injury mainly include strength, jump and COD tasks in a predominantly non-fatigued state [14–16]. From an injury prevention perspective, aerobic fitness [17] and particularly fatigue resistance between successive agility efforts also are relevant but still undervalued aspects that should consequently be considered in RTS scenarios.

The pathway to fatigue and injury has been reported to be similar, resulting in lower motor coordination and loss of movement efficiency [18]. There is evidence that fatigue may negatively affect joint kinematics in single-leg landing and cutting tasks [19, 20], especially under unanticipated conditions in female athletes [21, 22]. These altered movement strategies could predispose athletes to greater injury risk [23–25]. However, recent meta-analyses and reviews indicate that the overall effect of fatigue on performance as well as kinematic and kinetic variables is inconsistent [26–28]. One possible explanation could be the variable definitions of fatigue and different methodological approaches regarding the induction of fatigue. Furthermore, current fatigue protocols mostly involve pre-planned or non-specific tasks without additional cognitive loads mimicking less real-world scenarios [19]. Therefore, the development of ecologically valid fatigue protocols seems to be necessary in experimental settings, since athletes need to maintain constant motor control and agility performance even under sport-specific fatigued conditions [19, 29].

The SpeedCourt (SC) represents a novel method for training and testing multidirectional sprint performance, typical of game sports (i.e., sport-specific COD speed) [1]. The reliability of different SC protocols is sufficient [30]. The running directions of players on the SC platform are controlled by successively illuminating the different SC contact plates on a screen, enabling predefined or particularly random sprint sequences [1, 30]. The latter can be described as a mode of reactive agility since the players perform unplanned COD movements in response to a visual stimulus [1]. However, although the SC performance consists both of cognitive and physical factors, anticipation, pattern recognition and decision-making based on specific stimuli (e.g., opponent behaviour, changing game situations etc.) are missing. Consequently, SC actions can better be classified as agility-like movements.

There are multiple options for programming adequate fatigue protocols. The magnitude of exercise-induced fatigue highly depends on the manipulation and interaction of several loading variables such as number of sprint intervals, sprint distance per interval or the work/relief ratio (W:R). However, the acute fatigue responses to various SC sprint protocols have not yet been investigated. There are two studies that examined the effects of a three-week SC training program on speed and performance variables in young soccer players [1] and ACL deficient patients [31] using W:R ranging from 1:2 to 1:4, but both investigations did not report possible fatigue effects of the protocols analysed. In addition, considerable research has been carried out to investigate the post-exercise physiological responses of different high-intensity interval training (HIIT) formats. However, all HIIT running protocols were performed either straight-line or shuttle-based in predefined directions lacking cognitive loads in high density [32, 33].

Therefore, the aim of this study was to investigate the acute fatigue effects of different multidirectional agility-like SC sprint protocols on metabolic load, cognitive function and neuromuscular performance. It was hypothesised that all SC protocols induce relevant levels of fatigue, but a shorter relief duration in the W:R would cause a higher metabolic load and a greater decline in function and performance.

## Materials and methods

### Study design

**Fig. 1:**
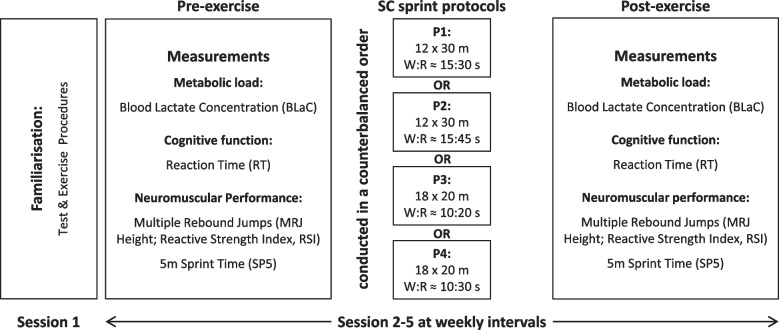
Schematic representation of the study design

We used a randomised crossover study design (Fig. 1) because the within-subject variation is less than the between-subject variation, which thus requires a smaller number of subjects. Using a computer-generated simple random sequence allocation (the SC protocol sequence was unique for each player), eighteen elite female soccer players of the 1st German Division completed a total of four volume-equated SC protocols differing in W:R and sprint distance travelled per interval (protocol 1, P1: 12 × 30 m, W:R = 1:2; protocol 2, P2: 12 × 30 m, W:R = 1:3; protocol 3, P3: 18 × 20 m, W:R = 1:2; protocol 4, P4: 18 × 20 m, W:R = 1:3). The specific protocol sequence was randomly drawn by the players before the start of the interventions. Pre- and post-exercise and in the following order, metabolic load was measured per blood lactate concentration, cognitive function per reaction time test, and neuromuscular performance containing a multiple rebound jump test and a 5 m linear sprint test. The SC sprint protocols were conducted on four different days, separated by a one-week washout-period, and took place at the same time of day to minimise diurnal variations in performance. We therefore did not expect potential carry-over effects from one week to the next. The soccer players were encouraged to use their maximum effort during the SC sprint exercise and within all measurements. Prior to the start of the session, a standardised dynamic warm-up was carried out including running ABC drills, dynamic stretching exercises as well as submaximal jump and sprint routines. In addition, one week before the onset of this study, there was a separate laboratory visit to sufficiently familiarise the players to the testing and training conditions.

All data was collected by an experienced sports scientist (PhD) and a bachelor’s student (B.Sc. cand.). Exclusion criteria were a severe injury of the lower limbs within the last six months before the start of the study (e.g. ACL injury), recovery from an acute muscle injury in the last six weeks before the start of the study, persistent pain, and an inability to train or compete. In each session, players wore the same shoes and clothing (i.e., club jersey). Subjects were advised to maintain their habitual diet and refrain from vigorous activity, especially for the lower extremity, at least 24 h before the start of the experimental procedures. This also applied to the consumption of alcohol and pain medication. Water was allowed to be drunk ad libitum in all sessions.

### Subjects

A convenient sample of eighteen elite female soccer players of the 1st German Divison volunteered to participate in this study (mean ± SD; age [yrs]: 23.1 ± 4.6, height [cm]: 167.9 ± 5.2, weight [kg]: 63.3 ± 6.6, BMI [kg ∙ m^2^]: 22.4 ± 1.5). All subjects were field players. Goal keepers were not considered because of their special game demands. The study took place before and during the pre-season preparatory period and in rare cases during the season in the movement laboratory (Athletikum Rhein Ruhr) at the BG Klinikum Duisburg. Training volume averaged at about eleven hours divided into four sessions per week. Within the season period the weekend training sessions were usually replaced by a competitive match. Before study onset, the players were informed about the goals, risks and procedures of the study and they all provided written informed consent. The study was approved by the local ethics committee (Ärztekammer Nordrhein, Düsseldorf, Germany; sequential number 2020224) and adheres to CONSORT guidelines. A sample size calculation via G*Power (version 3.1.9.4) was performed a priori and revealed that a minimum of sixteen subjects were required to significantly verify an effect size of Cohen´s *d* = 0.5. The following G*Power model was used: F-tests, ANOVA repeated-measures, within factors; *f* = 0.25, *α* = 0.05, (1-*ß*) = 0.80, number of measurements = 2, number of groups = 4, correlation among repeated measures = 0.8 [34]. During the course of the study, there were neither exclusions of participants nor adverse effects to the interventions.

### SC sprint protocols

The different sprint protocols were performed on the SC (Globalspeed GmbH, Hemsbach, Germany) which is a movement platform with the dimensions of about 5 × 5 m. It consists of nine contact plates embedded in the surface arranged in a chessboard pattern. The contact plates are connected with analogous user software and highlighted on a frontally-aligned flat screen with an interactive display of the SC platform illustrating the randomised running paths to the players. For each sprint sequence, the starting position was slightly behind the middle contact plate of the SC with the players facing the screen. After a three-second visual countdown (displayed on the screen), the players had to sprint at maximal effort to the first contact plate and after foot touch-down the next contact plate was visualised randomly on the screen.

The SC protocols were designed to intentionally induce fatigue and contained multiple COD movements or turns of various angles. All protocols were volume-equated and covered a total distance of 360 m that corresponds to the total distance travelled while sprinting during competition in elite female soccer players (160–650 m) [7]. The main differences between protocols were the applied W:R (1:2 vs. 1:3) and the sprint distance travelled per interval (20 vs. 30 m). The protocols 1 (P1) and 2 (P2) included twelve consecutive sprint intervals covering a distance of 30 m, in which the players had to run to ten contact plates as quickly as possible. The estimated sprint time was ≈15 s and the relief duration between intervals were 30 and 45 s corresponding to a W:R of 1:2 in P1 (W:R≈15:30 s) and a W:R of 1:3 in P2 (W:R≈15:45 s). In contrast, the protocols 3 (P3) and 4 (P4) included eighteen consecutive sprint intervals covering a distance of 20 m, in which the players had to touch seven contact plates with their foot as quickly as possible. The estimated sprint time was ≈10 s and the relief duration between intervals were 20 and 30 s corresponding to a W:R of 1:2 in P3 (W:R≈10:20 s) and a W:R of 1:3 in P4 (W:R≈10:30 s). All protocols contained one COD approximately every 3 m and the targeted mean sprint times of ≈15 s (P1 and P2) and ≈10 s (P3 and P4) were based on preliminary investigations.

## Measurements

### Sprint times

The sprint times of each interval was secured by the SC user software. We calculated the mean total time as well as mean split times of the first, second and final third of the different protocols.

### Metabolic load

Capillary whole-blood samples were taken from the hyperemised earlobe and analysed for blood lactate concentration (BLaC). BLaC was measured before and immediately after the different SC sprint protocols and was used to assess the metabolic load. Blood samples were taken with 20 µl capillaries, hemolysed in 1-ml microtest tubes and analysed enzymatic amperometrically by the SUPER GL3 (Dr. Müller Gerätebau GmbH, Hitado GmbH, Möhnesee, Germany).

### Cognitive function

A simple reaction time test was conducted before and after the different protocols. The test aimed to evaluate cognitive function according to stimulus processing speed. The players stood in front of the middle contact plate of the SC facing the screen. The contact plate was divided into a right and left half. One of the two halves randomly lit up on the screen, which the players had to touch with their foot as quickly as possible. As soon as the plate was activated, the next plate lit up using random delay (2–5 s) to minimize rhythmic anticipation. Reaction time (RT) was measured as the time elapsed between stimulus application and touching the contact plate. By this means, this measure is independent of physical performance qualities such as strength or power. The test included two sets with a total of ten repetitions each (five repetitions per side). The mean was taken for further analysis.

### Neuromuscular performance

A multiple rebound jump (MRJ) test and a 5 m linear sprint test were used to assess neuromuscular performance. Both tests were conducted before and after the different SC sprint protocols and were completed indoors on a laboratory tartan track. The MRJ test was carried out with an optical measurement system consisting of a transmitting and receiving infrared LED bar (OptoGait; Microgate, Bozen, Italy). The participants were advised to place their hands on their hips, and to perform repeated maximum vertical jumps for 15 s with reactive landing phases and ground contact times that should be as short as possible. Flight times and contact times were continuously collected. MRJ height was determined using the flight-time method (1/8 * g*t^2^). MRJ height and contact times were used to calculate the reactive strength index (RSI [Index] = MRJ height [m]/contact time [s]) [35]. The mean MRJ height and the mean RSI value of the five best RSI scores were taken for further analysis. Previously measured reliability scores for the MRJ were regarded as highly reliable (unpublished results; n = 38; RSI: Intra-class correlation coefficient, ICC = 0.91; typical error, TE = 0.13).

For the linear sprint test, 5 m sprint times (SP5) were recorded using a wireless single photocell system (Witty, Microgate, Bozen, Italy). Each sprint was initiated without a starting signal and from an individually chosen upright standing position 50 cm behind the first photocell. The participants performed two maximal sprints interspersed by 3 min of passive recovery. The mean SP5 was used for further analysis.

### Statistical analyses

Values are presented as mean ± SD. All statistical analyses were performed using JASP (version 0.16.4, Amsterdam, Netherlands). Before calculating any parametric tests, the sphericity was verified by the Mauchly-Test. A two-factor repeated-measures ANOVA was performed for the variables BLaC, RT, MRJ, RSI, and SP5 in order to determine differences between the measurement points (main effect for time), between the protocols (main effect for protocol) and for the changeover time in response to the different SC protocols (time x protocol interaction). A one-factor repeated-measures ANOVA was calculated for the different split times per protocol. If significance was found, Bonferroni-Holm post-hoc comparisons were performed. To allow a better interpretation of the results, effect sizes (Cohen´s *d*) were also calculated and values of 0.2, 0.5 and > 0.8 were considered small, medium, and large, respectively [36]. A *p*-value < 0.05 was considered statistically significant.

## Results

### Sprint times

There was a significantly improved performance in sprint time from the first to the second third in P4 (*p* = 0.017, *d* = 0.70), while the other split times of the different protocols were not significantly different (Table 1).

**Table 1:** Mean split sprint times and total sprint times of the different SC protocols

Mean sprint times [s]				
**Variable**	**P1**	**P2**	**P3**	**P4**
First third	14.82 ± 0.82	14.50 ± 1.06	10.58 ± 0.58	10.46 ± 0.44
Second third	14.46 ± 0.63	14.35 ± 0.89	10.33 ± 0.62	10.04 ± 0.41^a^
Final third	14.62 ± 1.06	14.32 ± 0.96	10.35 ± 0.66	10.19 ± 0.59
Total time	14.63 ± 0.65	14.39 ± 0.85	10.42 ± 0.47	10.23 ± 0.34

P1 = 12 × 30 m, W:R = 1:2, P2 = 12 × 30 m, W:R = 1:3; P3 = 18 × 20 m, W:R = 1:2, P4 = 18 × 20 m, W:R = 1:3

^a^significantly different to the first third of the same condition

### Metabolic load

There was a significant main effect for time (*p* < 0.001) and protocol (*p* < 0.001) in BLaC as well as a time x protocol interaction effect in BLaC (*p* < 0.001). Bonferroni-Holm post-hoc comparisons revealed significant higher BLaC values post-exercise in P1 (4.52 ± 1.83 mmol/L) compared to P2 (3.79 ± 1.83 mmol/L; *p* = 0.029, *d* = 0.74) and P4 (3.12 ± 1.83 mmol/L; *p* = 0.002, *d* = 1.06), whereas P3 (4.23 ± 1.69 mmol/L) elicited higher BLaC concentrations compared to P4 (*p* = 0.029, *d* = 0.74) following exercise cessation.

### Cognitive function

**Fig. 2:**
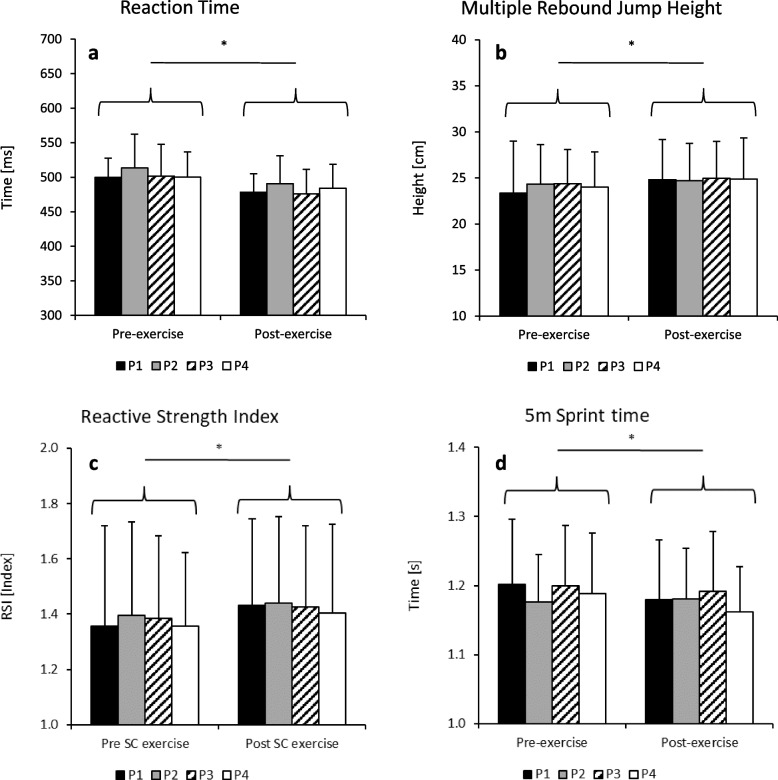
Effects of various SC sprint protocols on reaction time (**a**) and jump (**b**, **c**) and sprint performance (**d**).*denotes a significant main time effect. P1 = 12 × 30 m, W:R = 1:2; P2 = 12 × 30 m, W:R = 1:3; P3 = 18 × 20 m, W:R = 1:2; P4 = 18 × 20 m, W:R = 1:3

There was a significant main effect for time in RT with enhanced cognitive function after the SC sprint protocols (504 vs. 482 ms; *p* < 0.001, *d* = 1.95). There was no significant main protocol effect (*p* = 0.158, *d* < 0.5) or a significant time x protocol interaction effect (*p* = 0.596) in RS (Fig. 2a).

### Neuromuscular performance

Repeated-measures ANOVA revealed significant main time effects with improved performance post-exercise in MRJ height (24.0 cm vs. 24.8 cm; *p* = 0.004, *d* = 0.77), RSI (1.39 vs. 1.43; *p* = 0.007, *d* = 0.72) and SP5 (1.19 s vs. 1.17 s; *p* = 0.029, *d* = 0.56). There were no significant main protocol effects (*p* > 0.301) or significant time x protocol interaction effects (*p* > 0.304) in any of the neuromuscular performance variables (Fig. 2b-d).

## Discussion

This study aimed to evaluate the acute fatigue effects of different volume-equated agility-like SC sprint protocols, differing in W:R (1:2 vs. 1:3) and sprint distance per interval (20 m vs. 30 m), on metabolic load as well as on measures of cognitive and neuromuscular performance. We found significantly higher BLac values post-exercise in protocols that used a shorter (P1 and P3) compared to a longer relief duration (P2 and P4) at the same sprint distance, while total metabolic load was moderate across all protocols. Contrary to our initial hypothesis, none of the applied SC protocols induced fatigue on the cognitive level (reaction time) or on the physical level (i.e., jump and sprint performance) in elite female soccer players. Instead we surprisingly observed a significantly increased cognitive function and neuromuscular performance post-exercise. Irrespective of the outcome of this study, the present data may be useful as reference data for improved RTS decision-making for elite female soccer players.

### Metabolic load

In general, there was low exercised-induced metabolic fatigue reflected by BLaC values ranging from 3.12 to 4.52 mmol/L across all protocols. This indicates a contribution from the anaerobic glycolytic energy pathway to a lesser extent and suggests a metabolic balance near the lactate steady-state at the systemic level [37]. It has been reported that COD sprints are metabolically less demanding than linear sprints due to the low metabolic demand of the deceleration phase that may not be offset by the increased energy requirement of the reacceleration phase [38]. Since the players performed 7–10 CODs per interval during short-distance multidirectional sprinting, this could be a possible explanation.

Additionally, research indicate sex-dependent differences in metabolic and neuromuscular properties, with females reported to be more resistant to fatigue [39]. This may be related to a lower glycolytic enzyme activity and greater reliance on aerobic metabolism combined with a likely greater distribution of type I fibres compared to males, who have a higher initial power output and therefore a greater involvement of anaerobic glycolysis during repeated-sprint exercise [39]. This could also explain the moderate BLaC levels in our study. To compare our results with the current body of evidence is hard since we are not aware of any similar research. Most of the studies have primarily been carried out on male soccer players [40, 41] or used a different methodological approach (e.g., repeated sprint cycling) [42, 43]. In summary, the design of the applied SC protocols was not sufficient to induce metabolic fatigue in elite female soccer players.

There were significantly higher BLaC values post-exercise in protocols that used a shorter (P1 and P3) compared to a longer relief duration (P2 and P4) at a constant sprint distance. Contrary to this, we found no differences in BLaC levels between protocols that covered a longer (P1 and P2) or shorter sprint distance (P3 and P4) at a constant W:R. Thus, the major driver for the higher BLaC values in P1 and P3 seems to be the shorter relief duration as opposed to the manipulation of sprint distance travelled. It can be assumed that the shorter recovery time in P1 and P3 more strongly impair adequate phosphocreatine resynthesis that slightly taxes more anaerobic glycolysis to meet the energy demands of repeated sprinting [4]. This is supported by previous research showing that a gradual reduction of the relief duration (6 × 40 m repeated shuttle sprint test separated by 25/20/15 s recovery for each test) is associated with increasing BLaC values, at least in young male soccer players [44]. Based on the conditions of this study, it therefore can be assumed that female players need a shorter relief duration during repeated-sprint exercise (e.g., W:R = 1:1) because of a greater fatigue resistance in order to amplify blood lactate accumulation and metabolic fatigue. However, this needs to be confirmed in further research.

### Cognitive function

Irrespective of protocol design, there was an improved cognitive function post-exercise. None of the protocols induced mental fatigue associated with decreased stimulus processing speed (as measured by RT). Research indicates that acute exercise basically has an inverted U-shaped effect on the performance of a cognitive task with respect to exercise intensity. In particular, improvements in RT performance were usually observed at exercise intensities ranging from 40 to 60% of maximal oxygen uptake (VO2max) or below the lactate threshold, respectively [45]. This means that the aerobic metabolism is predominantly targeted without blood acidosis or accumulation of metabolic waste products [46]. In this study, we did not determine VO2max or the lactate threshold, since the intensity of all protocols was defined as all-out efforts. Considering the low exercise-induced metabolic load with BLaC values close to the lactate-steady state in all protocols, it can be assumed that the aerobic and phosphocreatine metabolism were the main contributors to energy supply during the repeated-sprint exercise, thus possibly explaining the increase in post-exercise RT performance.

The underlying mechanism has been linked to enhanced central noradrenergic activation, with a significant relationship between the concentration of plasma catecholamines and RT performance during moderate exercise [47]. However, we did not measure blood noradrenaline or adrenaline levels, so this remains a plausible speculation. Another possible factor for the improvement in post-exercise RT may be related to exercise specificity that induced an enhanced stimulus processing, since the players received the same visual stimuli during the repeated-sprint exercise and the RT test. Consequently, the applied SC protocols are capable to significantly improve RT performance, making them valuable for implementation in specific warm-up routines before training or competition in elite female soccer players, for example. To induce mental fatigue by means of deterioration in RT, it can be speculated that it is necessary to increase exercise intensity or the degree of post-exercise physical exertion [45]. During SC repeated-sprint exercise this could be achieved by increasing the sprint distance or the number of intervals as well as by decreasing the relief duration between intervals. Whether the manipulation of these variables has an effect on RT performance needs to be shown in future studies.

### Neuromuscular performance

There were significant increases in jump and sprint performance after completing all of the SC sprint protocols. From a metabolic view, contractile function seemed to be little affected due to the relatively low level of exercise-induced metabolic fatigue and the correspondingly low accumulation of metabolic by-products from anaerobic metabolism [48]. From a performance perspective, we also did not record any fatigue-related increases in split times from the first to the final third of the SC sprint protocols. However, this does not explain the increase in jump and sprint performance, but it does provide favourable conditions for post-activation performance-enhancing (PAPE) effects that rely on other mechanisms and typically peak between 5 to 10 min after the conditioning activity [49]. These potential PAPE effects may be associated with increased muscle temperature (i.e., temperature-sensitive ATPase reaction increases the rate of force development and contraction velocity), alterations in muscle water content (i.e., the resulting decrease in ionic strength increases muscle fibre force) and increased neural drive (i.e., increased spinal-level synaptic excitability heightens net motoneuron output, partially through enhanced motivation) [50]. It is likely that these and potentially other factors contributed to a composite effect.

The present results surprised us, as we primarily wanted to investigate the extent of neuromuscular fatigue induced by the different SC protocols. We did not expect a completely opposite and performance-enhancing effect. This makes a comparison to other studies difficult, since heavy strength or plyometric rather than repeated-sprint exercises are typically used as pre-conditioning activities to investigate their potential PAPE effects on jump or sprint performance [49, 51]. In this respect, it has been reported that the coupling of a biomechanically similar high-load and high-speed exercise during a so-called complex training program (e.g., alternating squats and countermovement jumps, CMJ) is likely to induce a PAPE effect in the neuromuscular system, resulting in increased power output during subsequent jump and sprint exercises [52, 53]. Since the repetitive short-distance sprints and reactive CODs of the SC protocols have similar biomechanical demands as the subsequent MRJs and linear sprints, the increase in performance could be explained through a possible PAPE effect.

In addition, one study investigated the potentiating effects of heavy squat exercise on CMJ performance immediately after the conditioning activity as well as before and after each of five match-sets in elite female volleyball players. In most cases the authors found a significantly higher CMJ performance between baseline and all other measurement times with CMJ heights being consistently higher in the experimental compared to the control group [54]. However, there also was an increased CMJ performance between baseline and match-set 3 and 5 in the control group, possibly suggesting that sport-specific movements themselves could elicit a PAPE effect. This may well also support our findings. In summary, all SC protocols induced an increase in jump and sprint performance that likely is associated with a PAPE effect. In order to cause fatigue with a relevant decline in neuromuscular performance, a further reduction of the recovery time between repeated-sprint efforts seems to be promising [55]. Future research will have to prove this assumption.

The different protocols were designed to intentionally induce fatigue under ecologically more valid conditions, similar to those of game sports. As already mentioned, we failed to induce fatigue effects with the existing protocols and surprisingly observed an increase in cognitive and neuromuscular performance post-exercise. Nevertheless, we believe that the protocols could still be useful for RTS decision-making. For example, if a female player undergoing RTS testing showed a decrease in performance after completing one of the protocols (in particular P1 or P3), this would possibly indicate a low resistance to fatigue and poor recovery ability between repeated-sprint efforts. As a result, it can be assumed that the tolerance to intense training and competition loads is limited and the risk of re-injury is increased.

Some methodological limitations are to be considered. Most of the players completed the testing and training procedures before and during the pre-season preparatory period. However, due to time and organisational reasons, some players carried out the test and training programs during the season. Consequently, we cannot rule out differences in fitness levels between players which may have affected our results. Additionally, the running paths were automatically generated in a randomized form by the SC software, corresponding to a sprint distance of either 20 or 30 m travelled per interval. However, due to technical reasons, these were only approximate values that deviated from the actual sprint distances covered. The mean variability in sprint distances travelled ranged from 4.5 to 6.5 m across all protocols, which resulted in total differences of 16 to 27 m between players. Therefore, small differences in exercise volume could have had an impact on our results. Finally, we did not record ratings perceived exertion (i.e., RPE) nor did we measure VO2max and blood-borne markers (e.g., creatine kinase) to verify our explanations from a mechanistic perspective. Body fat was also not recorded. However, this should be taken into account in future approaches.

## Conclusions

The SC protocols failed to induce fatigue in elite female soccer players, therefore these cannot be used for their intended purpose of testing players´ performance readiness under sport-specific fatigued conditions in RTS scenarios, however, the protocols may be useful as reference data for improved RTS decision-making. Regarding the development of ecologically more valid fatigue protocols, future research should investigate if a further reduction of the relief duration (e.g., W:R = 1:1) is beneficial.

## Data Availability

Due to maintaining control of further data usage, the datasets generated and analysed during the current study are not publicly available. In general, we are keen to share our datasets, which is why they are available from the corresponding author on reasonable request.

## Abbreviations

ANOVA: Analysis of variance
BLaC: Blood lactate concentration
CMJ: Counter movement jump
COD: Change of direction
MRJ: Multiple rebound jumps
PAPE: Post-activation performance enhancement
RSI: Reactive strength index
RT: Reaction time
RTS: Return to sports
SC: SpeedCourt
SD: Standard deviation
VO2max: Maximal oxygen uptake
W:R: Work/relief ratio
